# Social determinants of maternal self-rated health in South Western Sydney, Australia

**DOI:** 10.1186/1756-0500-7-51

**Published:** 2014-01-21

**Authors:** Katie J Morgan, John G Eastwood

**Affiliations:** 1 Child Health Targeted Support Services, Community Based Services, Women, Youth and Children, Canberra Hospital and Health Services, Health Directorate, ACT Government, ACT Australia; 2 School of Public Health and Community Medicine, University of New South Wales, Sydney, New South Wales 2052, Australia; 3 School of Women’s and Children’s Health, University of New South Wales, Sydney, New South Wales 2052, Australia; 4 School of Public Health, University of Sydney, Sydney, New South Wales 2006, Australia; 5 School of Public Health, Griffith University, Queensland 4222, Australia; 6 Department of Community Paediatrics, South Western Sydney Local Health District, Liverpool, New South Wales 1871, Australia

**Keywords:** Self-reported health, Maternal, Social epidemiology, Immigrants, Social disadvantage, Social exclusion

## Abstract

**Background:**

From 2000 a routine survey of mothers with newborn infants was commenced in South Western Sydney. The aim of this study is to examine the relationship of maternal self-rated health, as a measure of well-being, to various socio-demographic factors including measures of social capital, country of birth, financial status and employment.

**Results:**

The sample consisted of 23,534 mothers who delivered in South Western Sydney between 2004 and 2006. The data were collected as part of a routine post-partum assessment at 2–4 weeks postpartum. We examined the relationship of self-rated health with socio-demographic variables using binary logistic regression. Worse self-rated health was reported in 4% of women. Variables which were found to be significantly associated with worse self-rated health were: poor financial situation, public housing accommodation, fathers employment, no car access, unplanned pregnancy, maternal smoking, poor emotional and social support, and motherhood being more difficult than expected.

**Conclusion:**

We confirmed the importance of social disadvantage and social isolation as independent risk factors for poor self-reported health. The findings reported here provide further justification for public health interventions which increase support for socially excluded mothers and strengthen their connection to their community.

## Background

Self-rated health has been used as a global measure of quality of life [1] and as a predictor of mortality and morbidity with good retest reliability [2,3]. The predictive value has been shown to be consistent across age groups, genders socio-economic groups and different ethnic groups [2,4,5]. Factors that are known to be associated with self-reported health include: gender, income, education, unemployment, culture, place and health behaviours [6]. Layers and colleagues [6] also demonstrated that self-reported health is, in part, determined by reporting behaviour as reflected by knowledge, expectations and social context.

Maternal physical and psychological well-being during pregnancy, childbirth and early childhood contribute to improved outcomes for infants in the early childhood years and throughout life [7,8]. In our previous studies of maternal mental health we reported an association between self-reported health and postpartum depressive symptoms [9,10]. In those studies we had postulated that maternal self-reported health was an independent cause of maternal depression along with maternal expectation, unplanned pregnancy and measures of socioeconomic deprivation, neighbourhood environment, social capital and ethnic diversity. We did not, however, examine self-reported maternal health as a separate outcome.

There have been few studies of self-reported health during the pregnancy and early infancy. A study of pregnant women found that poor self-rated health was associated with a poor obstetric history. The authors proposed that the childbirth experience may have had long-term effects on the women’s emotional well-being, mental health and family stress [11]. Another study has found an association between low birth weight and a mother’s self-rated health [12].

Studies on mothers of young babies have found that young maternal age, full time employment, high income, low socio-economic status and lack of a partner were associated with poor self-rated health [13]. Good self-rated health on the other hand was associated with support from her husband. Predictors of parental stress including the number of young children have been found to be associated with poor self-reported mental health [14]. Social position was also important in predicting self-reported health in pregnant women [15]. Studies of self-rated health and social capital have produced conflicting results [16-21].

The aim of this study is to examine the relationship of maternal self-rated health, as a measure of well-being, to various socio-demographic factors including measures of social capital, country of birth, financial status and employment.

## Methods

The study reported here is a cross sectional study of (n = 23,534) mothers and their infants in South Western Sydney, New South Wales, Australia, between 2004 and 2006. The region experiences high levels of social disadvantage and migration. Measures of social disadvantage such as the Index of Relative Socioeconomic Disadvantage (IRSD) consistently show the region to be disadvantaged compared to other parts of Sydney and New South Wales. The region also has a large non-English speaking migrant population [22].

South Western Sydney Area Health Service (SWSAHS) as part of its routine initial assessment of mothers and babies in the first month post-partum collected data to be included in the Ingleburn Baby Information System (IBIS) database [23,24]. The IBIS questionnaire was completed for 23,534 mothers. There were no exclusions. In this study the IBIS survey was administered to non-English speaking mothers through interpreters. Ethics approval was granted from Sydney South West Area Health Service Human Research Ethics Committee and the UNSW Human Research Ethics Committee.

### Outcome variable

The outcome variable used in this study was self-rated health (SRH). Self-rated health can be interpreted as a global measure of quality of life [1] measuring health and well-being. All mothers included in the study were asked “in general how do you rate your own health?” Responses could be excellent, very good, good, fair or poor. For this study the responses were recoded to a dichotomous variable with excellent, very good and good being coded as better and fair and poor being coded as worse. This transformation to a dichotomous variable is consistent with previously reported studies.

### Exposure variables

The IBIS survey contains 45 items which are both clinical (e.g. weight) and parental self-report in nature (see Additional file 1). Socio-demographic exposure variables selected for analysis included: mother’s educational level, father’s employment, financial situation, accommodation, marital status, phone access, car access and Aboriginal or Torres Strait Island mother. Exposure variables selected as possible measures of social capital included: suburb duration, country of birth, regret about leaving the suburb (“If for some reason you had to leave this suburb would you be sorry to go?”), support network (“If you had any worries about your child, how many people do you feel you could turn to for help and support, not including health professionals?”), practical support (“Do you receive adequate practical support since the birth of the baby?), emotional support (“Have you been able to talk to someone about how you are feeling since the birth of the baby?”). Long suburb duration and regret about leaving a suburb are both measures of connectivity and strong social capital. Variables included as possible measures of domestic stress, and thus poor mental health, included: blended family (or reconstituted family), number of children under five years, household size, unplanned pregnancy, and poor practical support and emotional support. The variable selected that might be related to poor health behaviour was smoking in pregnancy. The IBIS question related to mother’s expectation of motherhood was selected as a measure of reporting behaviour. Mother’s expectation of motherhood was asked as: “Is being a mother what you expected”.

### Statistical analysis

Statistical analysis consisted of: 1) cross-tabulations; 2) unadjusted logistic regression, and 3) adjusted logistic regression. A final model in which non-significant odds ratios were excluded was also examined. Odds ratios and 95% confidence intervals will be presented for the logistic regression analyses. All analyses were undertaken using SPSS statistics 22.0 (SPSS, 2013).

## Results

During the study period there were 37,810 recorded births and 23,534 (62.2%) women had IBIS data collected at first post-natal visit. The mean age of the infant at time of assessment was 2.92 weeks. Frequencies of variables selected for analysis are shown in Table 1.

**Table 1:** Variables examined and chi square analysis

**Variable**	**Category**	**Total**	**% poor SRH**	**Pearson chi square**	***Df***	**P value**
Mothers education	Post yr 12	9969	3.47	35.45	4	<0.001
	Yr 12	4934	3.69			
	Yr 10	4878	4.51			
	Never/primary	583	6.69			
	Other	658	6.69			
Financial situation	Good	2488	2.33	120.95	2	<0.001
	Average	13353	3.88			
	Poor	1041	10.18			
Accommodation	Private	17673	3.68	63.40	2	<0.001
	Public	1160	8.36			
	Other	2435	3.74			
Father employment	Fulltime	14860	3.34	67.47	4	<0.001
	Casual/part time	960	6.04			
	Self emp	2345	3.11			
	Umemp	1134	5.91			
	Other	943	7.10			
Phone access	Yes	20948	3.90	8.68	1	0.003
	No	396	6.82			
Car access	Yes	16899	3.65	21.70	1	<0.001
	No/occas	4544	5.17			
Aboriginality	No	20557	4.00	3.40	1	0.065
	Yes	463	5.62			
Marital status	Married	16038	3.62	47.60	2	<0.001
	Single	1900	6.89			
	Partner	3668	4.12			
Blended family	No	17763	3.88	3.78	1	0.520
	Yes	3142	4.61			
Household size	1 to 5	18058	3.78	11.77	2	0.003
	6 to 10	3300	5.00			
	>10	123	5.69			
Children under 5	1	11049	4.01	10.45	3	0.150
	2	8122	3.77			
	3	1672	4.72			
	4 or more	246	7.32			
Planned pregnancy	Yes	14719	3.01	103.02	1	<0.001
	No	6738	5.91			
Maternal smoking	No	17666	3.69	31.99	*1*	<0.001
	Yes	3096	5.85			
Country of birth	Australia	12622	3.51	14.45	1	<0.001
	Other	8656	4.54			
Suburb duration	3 or more	11135	3.83	1.80	*2*	0.407
	2 years	3729	3.86			
	1 year or less	6269	4.23			
Emotional support	Yes	18825	3.37	251.57	1	<0.001
	No	1630	11.41			
Practical support	Yes	18540	3.46	76.68	1	<0.001
	No	2779	6.91			
Support network	3 or more	18101	3.44	81.54	1	<0.001
	Less than 3	3017	6.89			
Regret leaving	Yes	16706	3.57	33.36	1	<0.001
	No	3960	5.56			
Expectations of motherhood	As expected or better	16329	2.92	201.90	1	<0.001
	Worse than expected	4851	7.44			

When asked about their own health 20,890 (88.8%) of women reported better health and 865 (3.7%) reported worse health. Data were missing for the remainder of the women 1,779 (7.6%). The variables which were related to self-reported health in the Chi Square and univariate analysis were: low maternal education, poor financial situation, public housing accommodation, fathers unemployment, no phone access no car access, single marital status, household size 6–10, four or more children under five years, unplanned pregnancy, maternal smoking, not born in Australia, poor emotional and practical support, small support network, no regret at leaving a suburb and motherhood being more difficult than expected. Variables which were not associated included: family blending, number of children under 5 years, suburb duration, and Aboriginal status. The unadjusted and adjusted logistic regression results are shown in Table 2.

**Table 2:** Uni-variate (unadjusted) and multi-variable (adjusted) logistic regression

		**Unadjusted 95% CI**			**Adjusted 95% CI**		
**Variable**	**Category**	**OR**	**Lower**	**Upper**	**OR**	**Lower**	**Upper**
Mothers education	Post yr 12						
	Yr 12	1.07	0.89	1.28	0.96	0.76	1.21
	Yr 10	1.31	1.11	1.56	1.06	0.83	1.36
	Never/primary	1.99	1.42	2.81	1.03	0.60	1.77
	Other	1.99	1.44	2.76	1.10	0.65	1.86
Financial situation	Good						
	Average	1.69	1.29	2.23	1.50	1.10	2.05
	Poor	4.75	3.42	6.60	3.12	2.05	4.72
Accommodation	Private						
	Public	2.39	1.95	2.98	1.58	1.10	2.27
	Other	1.02	0.81	1.27	2.12	1.32	3.43
Fathers employment	Full time						
	Casual part-time	1.86	1.41	2.46	1.51	1.06	2.15
	Self emp	0.93	0.73	1.19	0.98	0.72	1.32
	Unempl	1.82	1.40	2.37	0.74	0.49	1.13
	Other	2.22	1.70	2.88	1.20	0.82	1.76
Phone access	No	1.80	1.21	2.68	1.36	0.67	2.75
Car access	No	1.44	1.23	1.68	1.29	1.01	1.66
Aboriginality	Yes	1.46	0.97	2.18	1.33	0.75	2.34
Marital status	Married						
	Single	1.97	1.62	2.40	1.21	0.92	1.56
	Partner	1.14	0.95	1.37	1.36	0.88	2.11
Blended family	No						
	Yes	1.12	1.00	1.44	1.17	0.89	1.55
Household size	1 to 5						
	6 to 10	1.34	1.13	1.59	1.12	0.90	1.54
	> 10	1.54	0.71	3.30	1.32	0.45	3.91
Children under 5	1						
	2	0.94	0.81	1.09	0.83	0.67	1.01
	3	1.19	0.93	1.52	0.88	0.622	1.23
	4 or more	1.89	1.16	3.08	1.06	0.52	2.17
Planned pregnancy	No	2.02	1.76	2.32	1.65	1.35	2.03
Maternal smoking	Yes	1.62	1.37	1.92	1.29	1.00	1.67
Born in Australia	No	1.31	1.14	1.50	1.15	0.94	1.41
Suburb duration	3 or more						
	2 years	1.01	0.83	1.22	1.24	1.00	1.55
	1 year or less	1.11	0.95	1.30	1.10	0.82	1.45
Emotional support	No	3.69	3.11	4.38	2.33	1.77	3.06
Practical support	No	2.07	1.76	2.45	1.02	0.77	1.34
Support network	Support network < 3	2.08	1.77	2.44	1.41	1.12	1.80
Regret leaving	No regret leaving suburb	1.59	1.36	1.86	1.02	0.81	1.23
Expectations of motherhood	Expectations worse than expected	2.68	2.33	3.08	2.12	1.78	2.62

The significant variables in the logistic regression with all variables were: poor financial situation, accommodation, father’s casual part-time employment, no car access, unplanned pregnancy, maternal smoking, no emotional support, a support network less than three, and motherhood being more difficult than expected (see Table 2). The reported odds ratios did not differ significantly in a parsimonious model that included only significant variables (p < 0.05).

## Discussion

The prevalence of poor self-rated health in this population was 4%. This is lower than reported in other studies. A Swedish study found that 92% of women at 2 months after birth reported good self-rated health [25]. Other studies have found a prevalence of poor self-rated health of between 5.2-15% [4,26,27]. Those studies were, however, general adult population studies and could be expected to differ from our population of women of child bearing age.

There are conflicting results in the literature regarding the association of self-rated health with social capital. Wen [20] reported no association between health and sense of community or community involvement, which duration in a suburb and regret leaving the suburb may also measure. In this study we considered support network size, suburb regret, suburb duration emotional and practical support to be possible indicators for social capital. Emotional support (“Have you been able to talk to someone about how you are feeling since the birth of the baby?”) and support network size (“If you had any worries about your child, how many people do you feel you could turn to for help and support, not including health professionals?”) were both associated with self-reported health. Our other indicators of social capital including suburb duration, suburb regret and adequate practical support were not shown in this study to be associated with self-reported health.

Motherhood expectation was associated with self-reported health in this study. The association of self-reported health to maternal expectations of motherhood is consistent with Beck’s meta-synthesis of 18 qualitative studies of postpartum depression which identified “incongruity between expectations and the reality of motherhood” as one of four perspectives of postnatal depression [28]. Whether a pregnancy is planned or not is associated with self-reported health in this study. This may be related to emotional support as mothers with unplanned pregnancies are less likely to have a partner who is supportive or present. An unplanned pregnancy that is also an unwanted pregnancy may also have an adverse effect on a woman’s mood and overall well-being.

As previously reported [18] maternal financial status was related to self-reported health. In our study the other measures of social position or social disadvantage that were associated with self-reported health, in the unadjusted regression, were maternal education (year 10 and no primary school), father’s employment (part-time and unemployed), accommodation (public housing), no phone access and no car access. Smoking was associated with self-reported health in this study as has been reported elsewhere [18,20]. The finding is consistent with known higher smoking rates among socially disadvantaged mothers.

Aboriginal and Torres Straight Island people in Australia experience significantly poorer health than the rest of the population within Australia [29]. In our study we found no association of self-reported health with being an Aboriginal or Torres Straight woman. This finding was in both the unadjusted and adjusted regression studies. While the self-rated health measure has been validated in a wide range of cultural groups it has not been specifically validated in the Australian Aboriginal population. It is also possible that the measurement of self-rated health among urban mothers is not related to the predominant causes of excess morbidity and mortality among Aboriginal and Torres Straight Island people.

The sample size (23,534) of this study of self-reported health among postpartum women is unique. The few similar studies of new mothers or pregnant women have sample sizes ranging from 878 to 5,368 women [13,15,25,30]. The sample was not, however, a complete population sample and there may have been a systematic bias. Mothers delivering at tertiary hospitals outside of the region are known to be less likely to receive their routine survey. The households not surveyed also included mothers who moved to “out of area” locations or mothers who declined the nurse first-visit offered. The population who refused an early childhood nurse visit may represent a particular socio-demographic sample. We were not able to analyse the characteristics of the mothers not surveyed. Missing data for most variables was less than 10 percent of cases. The exception to this was financial status for which data were missing in 26% of cases.

The cross-sectional design has limitations. The temporal direction of the relation between variables cannot be established with implications for drawing causal inference. The study used secondary data sources and the independent variables available for study were limited to those included in the IBIS self-report survey. The self-report nature of the survey is problematic with altered responses depending on mother’s mental state. Self-reporting may result in overestimated associations due to reporting bias and the underlying negative affectivity.

## Conclusion

Our study confirmed the importance of social disadvantage (difficult financial circumstances) and social isolation (lack of emotional and social support) as independent risk factors for poor self-reported health. These are both components of social exclusion. While the definition of social exclusion remains contested there is a common “understanding that social exclusion is not only about material poverty and lack of material resources, but also about the processes by which some individuals and groups become marginalised in society” [31]. Tsakloglou and Papadopoulos [32] grouped the indicators of social exclusion in to measures of income poverty, living conditions, necessities of life and social relations.

Given the growing evidence around life course effects, to improve health for children within the community, it is important to support their mothers both during and after their pregnancies as well as across the woman’s lifespan [33]. Ways of supporting women through pregnancy and the perinatal period may include sustained home visiting, mothers groups and telephone support [34-36]. The findings reported here provide further justification for public health interventions which increase support for socially excluded mothers and strengthen their connection to their community.

## Abbreviations

IRSD: Index of relative socioeconomic disadvantage; IBIS: Ingleburn baby information system; ROC: Receiver operating characteristics findings.

## Competing interests

The authors declare that they have no competing interests.

## Authors’ contributions

KM made the study designs, conceptualised the report, did the data analysis, data interpretation, and wrote the report. JGE made critical and technical contribution to the study design, analysis and report writing. All authors read and approved the manuscript.

## Supplementary Material

Additional file 1 South Western Sydney Area Health service I.B.I.S. paediatric baseline. Click here for file
