# Supplementary material for: Social determinants of maternal self-rated health in South Western Sydney, Australia
Source: BMC Res Notes. 2014 Jan 21;7:51. doi: 10.1186/1756-0500-7-51 (PMC3899616; doi:10.1186/1756-0500-7-51)
Supplement: Additional file 1 — South Western Sydney Area Health service I.B.I.S. paediatric baseline. [file 1756-0500-7-51-S1.zip › 1464543549747158_add1.pdf]

SOUTH WESTERN SYDNEY AREA HEALTH SERVICE
I.B.I.S. PAEDIATRIC BASELINE

Child's Surname (print)

Child's Given Names

1. **Gender:** ☐ Male ☐ Female

Mother's Surname (print)

Clinic Name

2. **Type of visit:** ☐ Clinic ☐ Home

3. **Attending:** ☐ Mother ☐ Father ☐ Grandparents ☐ Sibling 0-4 yrs ☐ >5yrs child ☐ Interpreter ☐ Other

Date Of Visit			Suburb	M.R.N.
Day	Month	Year		
10 0	Jan	00	0 0 0	0 0 0 0 0 0
20 1	Feb	01	1 1 1	1 1 1 1 1 1
30 2	Mar	02	2 2 2	2 2 2 2 2 2
3	Apr	03	3 3 3	3 3 3 3 3 3
4	May	04	4 4 4	4 4 4 4 4 4
5	Jun	05	5 5 5	5 5 5 5 5 5
6	Jul		6 6 6	6 6 6 6 6 6
7	Aug		7 7 7	7 7 7 7 7 7
8	Sep		8 8 8	8 8 8 8 8 8
9	Oct		9 9 9	9 9 9 9 9 9
	Nov			
	Dec			

Clinic	Staff Code	Date of Birth			B.W. grams	D.W. grams	Length cms	HC cms
		Day	Month	Year			.	.
			Jan					
0 0 0	0 0 0	10 0	Feb	1998	0 0 0 0	0 0 0 0	0 0 0	0 0 0
1 1 1	1 1 1	20 1	Mar	1999	1 1 1 1	1 1 1 1	1 1 1	1 1 1
2 2 2	2 2 2	30 2	Apr	2000	2 2 2 2	2 2 2 2	2 2 2	2 2 2
3 3 3	3 3 3	3	May	2001	3 3 3 3	3 3 3 3	3 3 3	3 3 3
4 4 4	4 4 4	4	Jun	2002	4 4 4 4	4 4 4 4	4 4 4	4 4 4
5 5 5	5 5 5	5	Jul	2003	5 5 5 5	5 5 5 5	5 5 5	5 5 5
6 6 6	6 6 6	6	Aug	2004	6 6 6 6	6 6 6 6	6 6 6	6 6 6
7 7 7	7 7 7	7	Sep	2005	7 7 7 7	7 7 7 7	7 7 7	7 7 7
8 8 8	8 8 8	8	Oct		8 8 8 8	8 8 8 8	8 8 8	8 8 8
9 9 9	9 9 9	9	Nov		9 9 9 9	9 9 9 9	9 9 9	9 9 9
			Dec					

4. **Hospital:** ☐ Bankstown ☐ Bowral ☐ Camden ☐ Campbelltown ☐ Fairfield ☐ Liverpool ☐ Other ________ 1 2 3 4 5

5. **Hearing:** ☐ Hearing risk identified **Referred:** ☐ AH ☐ Hospital ☐ Secondary Hearing **Hearing test:** ☐ Pass ☐ Review ☐ Fail

6. **Hearing blue book questionnaire:** ☐ Normal ☐ Refer 7. **Vision blue book questionnaire:** ☐ Normal ☐ Refer

8. **Feeding status:**

☐ **Breastfeeding**	☐ **Bottlefeeding**
☐ Exclusive	☐ Formula
☐ Fully	☐ Cows milk
☐ Partial	☐ Other
☐ Token	

9. **How is breastfeeding going?** ☐ Well ☐ With difficulty

10. ☐ Breast problems

11. ☐ Feeding problems

12. ☐ Solids commenced <4 months ☐ Solids commenced >4<6 months

13. **Weaned**

Weeks OR Months	
0 0	0 0
1 1	1 1
2 2	2 2
3 3	3 3
4 4	4 4
5 5	5 5
6 6	6 6
7 7	7 7
8 8	8 8
9 9	9 9

14. **If weaned, what affected your decision to stop breastfeeding?**
- ☐ Never wanted to breastfeed
- ☐ Baby was unsettled
- ☐ I am returning to work
- ☐ Care of other children
- ☐ Couldn't tell if my baby was getting enough milk
- ☐ Breastfeeding problems
- ☐ I did not have enough professional support
- ☐ Breastfeeding was a negative experience
- ☐ My husband did not support breastfeeding
- ☐ My family did not support breastfeeding
- ☐ Health professional advice
- ☐ Other

15. **Does the mother:**
- respond to the child's indications of discomfort? ☐ Yes ☐ No
- show the ability to comfort the child? ☐ Yes ☐ No
- enjoy close physical contact with the child? ☐ Yes ☐ No

16. **Since the birth of your baby, how much of the time did your baby seem:**

a)	To have trouble sleeping?	☐ All of the time	☐ Most of the time	☐ Some of the time	☐ A little of the time	☐ None of the time
b)	To be a demanding baby?	☐ All of the time	☐ Most of the time	☐ Some of the time	☐ A little of the time	☐ None of the time
c)	To be content?	☐ All of the time	☐ Most of the time	☐ Some of the time	☐ A little of the time	☐ None of the time
d)	To be a difficult feeder?	☐ All of the time	☐ Most of the time	☐ Some of the time	☐ A little of the time	☐ None of the time
e)	To be difficult to comfort?	☐ All of the time	☐ Most of the time	☐ Some of the time	☐ A little of the time	☐ None of the time
