# Supplementary material for: Social determinants of maternal self-rated health in South Western Sydney, Australia
Source: BMC Res Notes. 2014 Jan 21;7:51. doi: 10.1186/1756-0500-7-51 (PMC3899616; doi:10.1186/1756-0500-7-51)
Supplement: Additional file 1 — South Western Sydney Area Health service I.B.I.S. paediatric baseline. [file 1756-0500-7-51-S1.zip › 1464543549747158_add1_continue.pdf]

17. **Marital status:** ☐ Single ☐ Married ☐ Living with a partner

18. **Was this a planned pregnancy?** ☐ Yes ☐ No

19. **Household size:** ☐ One-five ☐ Six-ten ☐ >Ten

20. **Blended family:** ☐ Yes ☐ No

21. **How many children under five?**
☐ One ☐ Two ☐ Three ☐ Four or more

22. **Accommodation:**

Do you have a mortgage?	☐ Yes	☐ No
Do you own your own home?	☐ Yes	☐ No
Do you rent privately?	☐ Yes	☐ No
Do you rent public housing?	☐ Yes	☐ No
Are you living with parents?	☐ Yes	☐ No
Do you live in a caravan?	☐ Yes	☐ No
Do you live in a refuge?	☐ Yes	☐ No

23. **How long have you lived here in this suburb?**
☐ One year or less ☐ Two years ☐ Three or more years

24. **If for some reason you had to leave this suburb would you be sorry to go?**
☐ Yes, a lot
☐ Yes, a bit
☐ No, not much
☐ No, not at all

Current employment status:

25. **Mother:**	26. **Father:**
☐ Full time	☐ Full time
☐ Casual full time	☐ Casual full time
☐ Part time	☐ Part time
☐ Casual part time	☐ Casual part time
☐ Self employed	☐ Self employed
☐ Unemployed	☐ Unemployed
☐ Student	☐ Student
☐ Pension benefit	☐ Pension benefit
☐ Maternity leave	☐ Paternity leave
☐ Home duties	☐ Home duties
☐ Other	☐ Other

27. **Would you say that your financial situation is:**
Very difficult **Very good**
1 2 3 4 5 6 7 8 9 10

28. **Do you have access to a car?**
☐ Regularly ☐ Occasionally ☐ Never

29. **Can you ring out on your phone?** ☐ Yes ☐ No

30. **Are you of Aboriginal or Torres Strait Islander origin?**
☐ Yes ☐ No

31. **In which country were you born?** ☐ Australia ☐ Other

32. **What was the highest level of education you completed?**
☐ Never attended school, primary school
☐ School Certificate/Year 10/Intermediate/4th form
☐ HSC/Year 12/leaving 6th form
☐ TAFE Certificate/Diploma
☐ University, CAE or other tertiary institution degree
☐ Other

33. **In general how would you rate your own health?**
☐ Excellent ☐ Very good ☐ Good ☐ Fair ☐ Poor

34. **Do you smoke now?** ☐ Yes ☐ No

35. **If YES, how many are you smoking (most days of the week) now?**
☐ 1-5 cigs/day ☐ 6-15 cigs/day ☐ >15 cigs/day

36. **How long before you need your first cigarette in the morning?**
☐ Less than 30 minutes ☐ More than 30 minutes

37. **Drugs and alcohol:**
☐ Prescription drugs ☐ Recreational drugs ☐ Substance abuse

38. **Is being a mother what you expected?**
☐ Much more difficult than I expected
☐ More difficult
☐ What I expected
☐ Not as difficult as I expected
☐ Not at all difficult

39. **If you had any worries about your child, how many people do you feel you could turn to for help and support, not including health professionals?** ☐ None ☐ One ☐ Two ☐ Three ☐ >Three

40. **Do you receive adequate practical support (ie. help with the housework) since the birth of the baby?**
☐ Yes ☐ No ☐ Sometimes

41. **Have you been able to talk to someone about how you are feeling since the birth of the baby?** ☐ Yes ☐ No ☐ Sometimes

42. **Previous pregnancies:**
☐ Miscarriage ☐ Still birth ☐ Termination
☐ Death of a child ☐ Disability ☐ SIDS

43. **Edinburgh Scale:**
☐ PHN/FU ☐ Referred ☐ Medication
☐ Counselling
☐ Intervention declined ☐ Group work

Answer to question 10:
☐ 3 (Yes quite often)
☐ 2 (Sometimes)
☐ 1 (Hardly ever)
☐ 0 (Never)

0 0
1 1
2 2
3 3
4 4
5 5
6 6
7 7
8 8
9 9

44. **In general how would you rate your child's health?**
☐ Excellent ☐ Very good ☐ Good ☐ Fair ☐ Poor

45. **Referrals:**

	Medical	Social Support	Nursing/Allied Health
Primary:	☐ GP	☐ Volunteer	☐ Early Childhood Services
Secondary:	☐ Specialist	☐ Family Support	☐ Specialist
Tertiary:	☐ Hospital	☐ DOCS	☐ Residential Care

Consultation time
☐ 30-60 mins ☐ 65-90 mins ☐ >95 mins

Signature:____________________ **Date:**________________
